# Supplementary material for: A genome-wide association study identifies a novel susceptibility locus for the immunogenicity of polyethylene glycol
Source: Nat Commun. 2017 Sep 12;8:522. doi: 10.1038/s41467-017-00622-4 (PMC5595925; doi:10.1038/s41467-017-00622-4)
Supplement: Supplementary file 1 — Supplementary Information [file 41467_2017_622_MOESM1_ESM.pdf]

Reviewers' Comments:

Reviewer #1:
Remarks to the Author:
This is a slightly unusual but interesting and original study relating to drug safety. Production of antibodies to polyethylene glycol which is a constituent of some drugs limits their therapeutic efficiency. It is now established that this problem appears to relate to the existence of these antibodies prior to the start of drug treatment is some individuals. This study looks at a "healthy population" where levels of antibodies are measured from donated blood and then relates the presence of these antibodies to genotype in a GWAS. A positive association for IgM production with the immunoglobulin heavy chain locus is reported. This also replicates in another cohort. However, no association with IgG production was found. The absence of any patient data is a limitation.

Specific comments

1. No imputation appears to have been done. This would have increased the chance of detecting a positive signal for IgG so why not do this?

2. Was the top SNP also more common in the individuals positive for both IgG and IgM?

3. It would be helpful to include information about the frequency of the top SNP in other ethnic groups.

4. Did age and gender affect the incidence of IgG and IgM positivity?

Reviewer #2:
Remarks to the Author:
Reviewer Critique

In their study, entitled "A genome-wide association study identifies a novel susceptibility locus for the immunogenicity of polyethylene glycol (PEG)", Dr. Chang and colleagues performed genome-wide association studies for anti-PEG IgM and IgG responses in Han Chinese with 177 and 140 individuals, defined as positive for anti-PEG IgM and IgG responses, respectively, and with 492 subjects without either anti-PEG IgM or IgG as controls. The authors validated the association results in a replication cohort, consisting of 84 and 103 subjects with anti-PEG IgM and anti-PEG IgG, respectively, and 277 controls. The authors identified the immunoglobulin heavy chain (IGH) locus to be associated with anti-PEG IgM response at genome-wide significance (P = 1.67 × 10-15) and concluded that their finding may provide novel genetic markers for predicting the immunogenicity of PEG and efficacy of PEGylated therapeutics. While this study is of potential interest to the readership of Nature Communications, the cohort size is small and the study as such is underpowered for GWAS. I have the following comments:

Comments:
1. How do the authors validate that the GWAS association observed is not a non-specific association between IgM and the IGH locus (and potentially the IGHV gene).
2. It would be important to run IgM as a quantitative trait to see if there is at least nominal association observed with the IGH locus in the cases; it would also be of interest to run IgM as a quantitative trait in control (more as acute phase reactant) to ensure specificity of the results; if association is also observed in the controls, this would raise questions about the specificity of the association results to the anti-PEG IgM phenotype in the cases.
3. The effect sizes are strong and the allelic expression effects are impressive; in the Genotype-Tissue Expression (GTEx) database of expression quantitative trait locus (eQTL) analysis, is there

B-cell (plasma cell) specific association observed in the GTEx data? This would be anticipated from such a clonal finding.
4. Are the case subjects with anti-PEG IgM antibodies more prone to develop IgM responses?
5. Are the controls used shared for discovery and replication (same # of subjects) which is not optimal or are they independent.

Reviewer #3:
Remarks to the Author:
The authors present a GWAS of PEG immunogenicity, which is of interest given its usage to improve bioavailability in a number of drugs. They discover and replicate an association in the IGH locus. The methods and data are all high quality. I have only minor concerns:

1. I'm not sure I understand the analysis in Figure 3A. I think the authors have taken the genotypes as a dependent variable and compared the case/control ratio as an independent variable? But this is basically the same as the initial analysis of treating case/control status as a dependent and genotype as independent. In other words, if the genotype frequencies are different between cases and controls, then by definition the case/control ratio is different between genotypes. I would instead focus on the Figure 3B analysis, which brings new data (on absolute levels).

2. Could the authors comment on why they might've found an association for IgM, but not IgG?

3. In the legend of Fig 2, the colors are said to be LD measured by D', but the inset in the figure says r2. I think it's r2, but this should be consistent.

4. In Fig 2 it would be useful to connect via vertical dashed lines the discovery and joint p-values for the same SNPs to make it clear how they match up.

5. On line 97, the authors declare a genome-wide significant threshold of 8.97e-8. I'm not sure where this comes from, but I'd suggest they use the widespread threshold of 5e-8. This doesn't change the results, and will be more familiar to readers familiar with GWAS literature.

6. The sentence on lines 53-4 ("Until recently...") is confusing, as it suggests that immunogenicity is no longer controversial. I suggest rephrasing for better clarity.

Thanks for Reviewers' comments and suggestions. We have greatly increased the sample size in our replication cohort, from the original 84 and 103 subjects with anti-PEG IgM and anti-PEG IgG, respectively, and 277 controls, to 211 and 192 subjects with anti-PEG IgM and anti-PEG-IgG, respectively and 596 controls. The increase of sample size further substantiates our observation that anti-PEG IgM response, but not anti-PEG IgG response, is associated with the immunoglobulin heavy chain (IGH) locus.

Response to Reviewers' comments:

<u>Reviewer #1 (Remarks to the Author):</u>

This is a slightly unusual but interesting and original study relating to drug safety. Production of antibodies to polyethylene glycol which is a constituent of some drugs limits their therapeutic efficiency. It is now established that this problem appears to relate to the existence of these antibodies prior to the start of drug treatment is some individuals. This study looks at a "healthy population" where levels of antibodies are measured from donated blood and then relates the presence of these antibodies to genotype in a GWAS. A positive association for IgM production with the immunoglobulin heavy chain locus is reported. This also replicates in another cohort. However, no association with IgG production was found. The absence of any patient data is a limitation.

Specific comments

1. No imputation appears to have been done. This would have increased the chance of detecting a positive signal for IgG so why not do this?

Response: Thank you for the suggestion. For the purpose of having more associated evidence of an anti-PEG antibody response for IgG, genome-wide imputation was carried out by IMPUTE2 with the 1000 Genomes reference panel. Other than the top SNPs in the original GWAS, 27 imputed SNPs also showed marginal association with anti-PEG IgG level (*P*-value less than 10^{-5}) in the GWAS with the imputed data. These SNPs were selected for further validation and replication. However, none of these SNPs showed association with the anti-PEG IgG levels in the replication cohort (all *P*-value > 0.05).

Chr	dbSNP	GWAS Trend *P*	Replication Trend *P*	Joint Trend *P*

1	rs556198	9.37E-06	2.01E-01	1.77E-02
2	rs12712548	4.18E-08	7.99E-01	4.23E-05
2	rs71437595	4.18E-08	8.35E-01	3.80E-05
2	rs34029544	4.63E-08	4.07E-01	4.10E-04
2	rs6755353	5.16E-08	3.15E-01	4.48E-04
2	rs2373305	7.94E-08	3.04E-01	4.04E-04
2	rs12329319	8.41E-08	2.28E-01	3.72E-04
2	rs12469089	9.12E-08	2.62E-01	3.74E-04
2	rs13027679	9.12E-08	3.70E-01	3.89E-04
2	rs2373306	9.12E-08	2.76E-01	4.05E-04
2	rs11899949	9.5E-08	2.65E-01	3.58E-04
2	rs1946535	1.52E-07	2.80E-01	3.95E-04
2	rs868443	2.74E-07	4.35E-01	4.64E-04
2	rs934223	2.79E-07	3.95E-01	4.78E-04
2	rs885087	2.87E-07	4.35E-01	4.64E-04
2	rs13412507	8.73E-06	3.96E-01	3.89E-04
2	rs35664296	9.89E-06	2.79E-01	4.12E-04
4	rs60976364	7.64E-06	3.54E-01	6.19E-04
6	rs113854459	3.43E-06	1.41E-01	1.42E-02
6	rs12191160	4.49E-06	1.26E-01	1.76E-02
6	rs77137655	4.49E-06	1.26E-01	1.76E-02
6	rs7775619	4.49E-06	1.26E-01	1.76E-02
6	rs9470054	4.49E-06	1.26E-01	1.76E-02
8	rs1900815	9.12E-06	4.45E-01	3.99E-03
8	rs4909327	9.12E-06	4.45E-01	3.99E-03
13	rs3920613	8.81E-06	3.93E-01	1.81E-04
21	rs79714376	9.39E-06	9.05E-01	1.19E-03

2. Was the top SNP also more common in the individuals positive for both IgG and IgM?

Response: In the joint analysis for both anti-PEG IgG and IgM, none of these top SNPs in the *IGH* locus showed significant associations (all with *P*-values > 0.05). This result showed that the risk alleles for the top SNPs were not more common in the individuals positive for both IgG and IgM.

Chr	dbSNP	Trend *P*
14	rs10143619	0.6214
14	rs7154133	0.4653
14	rs8007516	0.3576

14	rs61999179	0.3769
14	rs7160708	0.4065
14	rs2157624	0.3935
14	rs12590237	0.2454

3. It would be helpful to include information about the frequency of the top SNP in other ethnic groups.

Response: Thank you for the suggestion. According to 1000 Genomes reference, the G allele frequencies of the top SNP rs12590237 are 0.39, 0.32, 0.41, and 0.34 for the African, American, East Asian, and European groups respectively, and similar to our control group (RAF controls = 0.388). The similar allele frequency of the top SNP across the different ethnic groups suggested that SNP rs12590237 could be a universal biomarker for prediction of anti-PEG IgM response among different ethnic groups. We have included the above information in lines 180-5.

4. Did age and gender affect the incidence of IgG and IgM positivity?

Response: According to our previous study (Chen et al., 2016), the incidence of anti-PEG IgM and IgG was significantly greater in females as compared to males. The prevalence of anti-PEG IgG is inversely correlated with age. By contrast, the incidence of anti-PEG IgM was not significantly associated with age.

Ref: Chen, B.M. *et al.* Measurement of pre-existing IgG and IgM antibodies against polyethylene glycol in healthy individuals. *Anal. Chem.* **88**, 10661-10666 (2016).

Reviewer #2 (Remarks to the Author):

Reviewer Critique

In their study, entitled "A genome-wide association study identifies a novel susceptibility locus for the immunogenicity of polyethylene glycol (PEG)", Dr. Chang and colleagues performed genome-wide association studies for anti-PEG IgM and IgG responses in Han Chinese with 177 and 140 individuals, defined as positive for anti-PEG IgM and IgG responses, respectively, and with 492 subjects without either anti-PEG IgM or IgG as controls. The authors validated the association results in a replication cohort, consisting of 84 and 103 subjects with anti-PEG IgM and anti-PEG IgG, respectively, and 277 controls. The authors identified the immunoglobulin heavy chain (IGH) locus to be associated with anti-PEG IgM response at genome-wide significance ($P = 1.67 \times 10^{-15}$) and

concluded that their finding may provide novel genetic markers for predicting the immunogenicity of PEG and efficacy of PEGylated therapeutics. While this study is of potential interest to the readership of Nature Communications, the cohort size is small and the study as such is underpowered for GWAS.

Response: Thank you for the comments. We have greatly increased the sample size in our replication cohort, from the original 84 and 103 subjects with anti-PEG IgM and anti-PEG IgG, respectively, and 277 controls, to 211 and 192 subjects with anti-PEG IgM and anti-PEG-IgG, respectively and 596 controls. The current sample sizes for the two-stage GWAS now reach a statistical power of 99% in the joint analysis, with the most significant SNP, rs12590237 having P value of 2.23×10^{-22} (greater than the original $P = 1.67 \times 10^{-15}$); odds ratio (OR) = 2.36. The power calculation was performed using CaTS (Skol et al. 2006) with parameters estimated from the current sample: prevalence of the trait = 20%, risk allele frequency in case = 60%, and relative risk = 2.3. The increase of sample size further substantiates our observation that anti-PEG IgM response, but not anti-PEG IgG response, is associated with immunoglobulin heavy chain (IGH) locus.

Ref: Skol A.D. *et al.* Joint analysis is more efficient than replication-based analysis for two-stage genome-wide association studies. *Nat. Genet.* 38, 209-213 (2006).

Comments:

1. How do the authors validate that the GWAS association observed is not a non-specific association between IgM and the IGH locus (and potentially the IGHV gene).

Response: As shown in Figure 3, the incidence and concentrations of anti-PEG IgM was significantly greater in homozygous and heterozygous risk alleles of IGHV polymorphism. This result suggests that IGHV polymorphism is directly associated with production of anti-PEG IgM and further validates the association between IgM and the IGH locus in GWAS analysis.

2. It would be important to run IgM as a quantitative trait to see if there is at least nominal association observed with the IGH locus in the cases; it would also be of interest to run IgM as a quantitative trait in control (more as acute phase reactant) to ensure specificity of the results; if association is also observed in the controls, this would raise questions about the specificity of the association results to the anti-PEG IgM phenotype in the cases.

Response: Thanks for the suggestion. We have performed linear regression analysis with IgM as a quantitative trait in the cases with positive anti-PEG IgM. The results

revealed that concentration of anti-PEG IgM was significantly correlated to risk allele G genotype of the top SNP rs12590237 in the *IGH* locus (P = 0.026). However, the concentration of anti-PEG IgM in most controls was close to zero and could not be assessed by linear regression analysis.

3. The effect sizes are strong and the allelic expression effects are impressive; in the Genotype-Tissue Expression (GTEx) database of expression quantitative trait locus (eQTL) analysis, is there B-cell (plasma cell) specific association observed in the GTEx data? This would be anticipated from such a clonal finding.

Response: Thanks for the comments. In the Genotype-Tissue Expression (GTEx) database of expression quantitative trait locus (eQTL) analysis (GTEx Consortium, 2015), the risk allele of the top SNP rs12590237 correlated with lower expression of *IGHV4-39* in the whole blood and the risk allele of a significantly associated SNP (rs7154133) correlated with lower expression of *IGHV3-53* in the spleen and whole blood. However, there were no B-cell (plasma cell) specific associations with these top SNPs in the GTEx data.

Ref: GTEx Consortium. The Genotype-Tissue Expression (GTEx) pilot analysis: Multitissue gene regulation in humans. *Science* **348**, 648-660 (2015).

4. Are the case subjects with anti-PEG IgM antibodies more prone to develop IgM responses?

Response: The healthy subjects in this study were randomly selected from the Taiwan Han Chinese Cell and Genome Bank in Taiwan. All of the subjects were in a de-linked fashion, and the clinical phenotypes of these subjects could not be tracked. Therefore, we could not confirm whether the case subjects with anti-PEG IgM antibodies are more prone to develop IgM responses.

5. Are the controls used shared for discovery and replication (same # of subjects) which is not optimal or are they independent.

Response: We used two independent cohorts for the genome-wide association study in the discovery and replication stages. The numbers of controls without either anti-PEG IgM or IgG are 492 and 596 for discovery and replication stages, respectively (Supplementary Table 2). These controls were shared for IgM and IgG analyses, but independent/non-overlapped for the discovery and replication analyses.

<u>Reviewer #3 (Remarks to the Author):</u>

The authors present a GWAS of PEG immunogenicity, which is of interest given its usage to improve bioavailability in a number of drugs. They discover and replicate an association in the IGH locus. The methods and data are all high quality. I have only minor concerns:

1. I'm not sure I understand the analysis in Figure 3A. I think the authors have taken the genotypes as a dependent variable and compared the case/control ratio as an independent variable? But this is basically the same as the initial analysis of treating case/control status as a dependent and genotype as independent. In other words, if the genotype frequencies are different between cases and controls, then by definition the case/control ratio is different between genotypes. I would instead focus on the Figure 3B analysis, which brings new data (on absolute levels).

Response: The genome-wide association study has shown that risk allele G genotype of the top SNP rs12590237 was significantly correlated with higher prevalence of anti-PEG IgM. However, Figure 3A clearly shows that the incidence of anti-PEG IgM was significantly greater in G/G and G/C genotypes as compared to the C/C genotype. This result is important and implies the dominant inheritance effect in the anti-PEG IgM response.

2. Could the authors comment on why they might've found an association for IgM, but not IgG ?

Response: IgM memory B cells have been described with different function and development pathways to the switched memory B cells (IgG+ or IgA+ B cell) (Pauli et al., 2011). In addition, IgM memory subsets also showed with different origins as compared to the switched memory B cells because of highly significant differences in their Ig heavy chain (IGHV) repertoires (Wu et al., 2010). In our study, we clearly demonstrated that a specific population with higher allele frequency of specific SNPs in the *IGH* locus was prone to produce anti-PEG IgM only instead of anti-PEG IgG. We suggest that this kind of anti-PEG IgM antibodies might be produced from IgM memory B cells and have different origin of IGHV repertoires from anti-PEG IgG. Therefore, these GWAS significant SNPs were only associated with this specific group of healthy subjects with anti-PEG IgM only, but not for IgG.

Ref:
1. Pauli, N.T., Henry Dunand, C.J. & Wilson, P.C. Exploiting human memory B cell heterogeneity for improved vaccine efficacy. *Front Immunol.* **2**, 77 (2011).
2. Wu, Y.C. *et al.* High-throughput immunoglobulin repertoire analysis distinguishes

between human IgM memory and switched memory B-cell populations. *Blood* **116**, 1070-1078 (2010).

3. In the legend of Fig 2, the colors are said to be LD measured by D', but the inset in the figure says r2. I think it's r2, but this should be consistent.
Response: Thanks for the suggestion. As shown in the legend of Fig 2, we changed the statement for LD measurement from D' to r^2.

4. In Fig 2 it would be useful to connect via vertical dashed lines the discovery and joint p-values for the same SNPs to make it clear how they match up.
Response: Thanks for the suggestion. As shown in Fig 2, we added vertical dashed lines to connect the same SNPs in discovery and joint stage.

5. On line 97, the authors declare a genome-wide significant threshold of 8.97e-8. I'm not sure where this comes from, but I'd suggest they use the widespread threshold of 5e-8. This doesn't change the results, and will be more familiar to readers familiar with GWAS literature.
Response: The genome-wide significant *P* value was calculated according to the Bonferroni correction for multiple-comparison test. After stringent quality control filtering, 557,394 SNPs were analyzed in the discovery stage (Supplementary Table 3). Therefore, using a nominal α=0.05 on the 557,394 SNPs results in a genome-wide significance threshold of 8.97e-8.

6. The sentence on lines 53-4 ("Until recently...") is confusing, as it suggests that immunogenicity is no longer controversial. I suggest rephrasing for better clarity.
Response: Thanks for the suggestion. We have changed the original sentence to the following one in line 73-4.
Original: Until recently, the non-immunogenicity of PEG is somewhat controversial due to the occurrence of anti-PEG antibodies in animal models immunized with PEGylated proteins and in patients treated with PEGylated biopharmaceuticals.

Modified: PEG has historically been considered to be poorly immunogenic but recent studies have demonstrated the occurrence of anti-PEG antibodies in animal models immunized with PEGylated proteins and in patients treated with PEGylated biopharmaceuticals.

Reviewers' Comments:

Reviewer #1:
Remarks to the Author:
I believe that the authors have dealt adequately with my suggested changes and those of the other reviewers. No further comments.

Reviewer #2:
Remarks to the Author:
The authors have been responsive to the critique raised and have addressed the reviewer comments and incorporated new results into the revised manuscript and notably improved on study power. I have no further comments on the revised manuscript.
